# Low temperatures can promote cyanobacterial bloom formation by providing refuge from microbial antagonists

**DOI:** 10.3934/microbiol.2018.2.304

**Published:** 2018-04-16

**Authors:** Thomas Rohrlack

**Affiliations:** Norwegian University of Life Sciences, Faculty for Environmental Sciences and Natural Resource Management, Postbox 5003, NO-1432 Ås, Norway

**Keywords:** cyanobacteria, bloom formation, thermal refuge, *Rhizophydium*, *Nassula*

## Abstract

Freshwater cyanobacteria are prone to a wide range of highly potent microbial antagonists. Most of these exploit their prey in a frequency-dependent manner and are therefore particularly well suited to prevent any accumulation of cyanobacteria. Mass developments of cyanobacteria, the so-called blooms, should therefore be rare events, which is in striking contrast to what we actually see in nature. Laboratory experiments of the present study showed that the temperature range 5.8–10 °C forms a thermal refuge, inside which the cyanobacterium *Planktothrix* can grow without being exploited by two otherwise highly potent microbial antagonists. In nature, access of *Planktothrix* to this refuge was associated with positive net growth and a high probability of bloom formation, confirming that refuge temperatures indeed allow *Planktothrix* to grow with a minimum of biomass loss caused by microbial antagonists. Contact to higher temperatures, in contrast, was associated with decreases in net growth rate and in probability of bloom formation, with population collapses and with the occurrence of parasite infection. This is in agreement with the finding of laboratory experiments that above 10 °C exploitation of *Planktothrix* by multiple microbes increases in a temperature-dependent manner. Taken together, above findings suggest that temperature modulates the microbial control of natural *Planktothrix* populations. Low temperatures form a thermal refuge that may promote *Planktothrix* bloom formation by shielding the cyanobacterium from otherwise highly potent microbial antagonists.

## 1. Introduction

The widespread occurrence of cyanobacterial mass developments, the so-called blooms, in freshwater systems is an ecological mystery that has yet to be fully resolved. Cyanobacteria are without doubt among the most successful phytoplankton groups when it comes to competition for growth resources and to minimizing loss of biomass due to zooplankton grazing [1],[2]. However, cyanobacteria are also prone to a vast range of potent microbial antagonists, including phages, lytic bacteria, heterotrophic flagellates and microparasites [3],[4]. These typically exploit their prey in a frequency-dependent manner [5], which means that the buildup of biomass that precedes a bloom greatly increases the risk of cyanobacteria of being devastated by microbial antagonists well before the actual bloom can develop. Cyanobacterial blooms should therefore be unlikely events, which is in striking contrast to what we actually see in nature [1],[2].

In response to being exploited by microbes, cyanobacteria have evolved several defense mechanisms. Examples include the CRISPR/Cas system that can confer acquired immunity to cyanophages [6], the chemical antiparasite defense of *Planktothrix* [7] and the behavioral defense of *Phormidium autumnale* that allows the cyanobacterium to escape herbivorous ciliates [8]. Another way of limiting the impact of microbial antagonists is intraspecific diversification of existing defense systems, which also seems common in cyanobacteria [3],[9],[10]. Yet, microbial antagonists of cyanobacteria often more than match their prey's adaptability and so many cyanobacterial defense mechanisms are met by microbial counter defense mechanisms [7],[11],[12]. This setting is likely to result in antagonistic coevolution [13] with so far unknown consequences for cyanobacterial bloom formation.

The outcome of antagonistic relationships is determined by the antagonists' properties and the environment that the antagonists live in [14]. The modulating effect of the environment can result in environmental refuges, which are ranges of environmental conditions that allow one part of an antagonistic relationship to reproduce without being exploited by the other part. Environmental refuges may, for example, arise from different tolerance ranges of the antagonists for temperature [15],[16], salinity [17], light [18], and nutrients [19]. A typical example is found in the relationship of the diatom *Asterionella formosa* and its fungal parasite *Zygorhizidium planktonicum*. The impact of the parasite is temperature-dependent, with temperatures between 0 and 1–2 °C allowing the diatom to grow without or nearly without infection [20],[21]. A 30-years study in Lake Maarsseveen [22] confirmed that this temperature range forms a thermal refuge, which promoted *A. formosa* spring blooms in years with cold winters. Warm winters, in contrast, resulted in low *A. formosa* densities with constant infection.

Inspired by these findings, I hypothesize that thermal refuges can promote bloom formation in cyanobacteria as well. As mentioned, cyanobacteria are exploited by a wide range of microbial antagonists [3],[4]. Some of these antagonists including cyanophages find their cyanobacterial prey by chance and may thus be most effective at a high cyanobacterial density, i.e., at the final stage of bloom formation [23]. Others such as zoosporic parasitic fungi [24],[25] and herbivorous ciliates [26] actively search for their prey. These microbes may thus be able to suppress cyanobacterial bloom formation at an early stage, when cyanobacterial density is still low. When assessing the potential of thermal refuges to promote bloom formation, one must also consider that a given cyanobacterial population might be exploited by multiple microbial antagonists at a time. If so, a microbial antagonist that is inhibited by unfavorable temperatures may be replaced by other, less temperature sensitive microbes. All in all, one can assume that thermal refuges can only promote bloom formation if these refuges protect a given cyanobacterial population at least from all local microbial antagonists that actively search for their prey.

In the present study I tested whether such thermal refuges actually exist and whether they indeed promote cyanobacterial bloom formation in nature. The focus of the study was on the Norwegian Lake Steinsfjorden that frequently experiences metalimnetic blooms of the cyanobacterium *Planktothrix* despite the presence of two highly potent microbial antagonists, the zoosporic fungal parasite *Rhizophydium megarrhizum* and the herbivorous ciliate *Nassula ornata*. Laboratory experiments were conducted to identify thermal refuges that protect *Planktothrix* against both microbes. These data were then combined with already existing monitoring data from Lake Steinsfjorden to retrospectively evaluate the role of thermal refuges in nature.

## 2. Materials and methods

### 2.1. Study area and study organisms

Situated north of Oslo, Lake Steinsfjorden is 20 m deep and it covers an area of 7.8 km^2^. The lake has a mean water residence time of 4.6 years [27]. Since systematic monitoring started in the early 1970s, annual means of total phosphorus and total nitrogen concentrations have remained stable at about 12 and 300 µg l^−1^, respectively, and summer blooms of *Planktothrix* in the lake's metalimnion (thermocline) have been common [27],[28]. Growth of *Planktothrix* in the lake is controlled by irradiance and temperature rather than by nutrients [28].

*Planktothrix* is very efficient in minimizing the loss of biomass. It possesses a gas vesicle based buoyancy regulation system, which prevents sedimentation, while at the same time allowing the cyanobacterium to actively move into water layers with favorable growth conditions [29]. The latter also minimizes the risk of nutrient limitation. The formation of long filaments limits zooplankton grazing, especially in lakes like Steinsfjorden that are dominated by small *Daphnia* and *Bosmina* species [27],[30]–[32]. However, microscopic and genetic analyses of environmental samples showed that the Steinsfjorden *Planktothrix* population is preyed on by *R. megarrhizum* and *N. ornata* [10],[33], which is a typical situation in *Planktothrix* populations forming metalimnetic blooms [34],[35]. Other microbial antagonists of *Planktothrix* were not found, although the methods used were not designed to detect relevant cyanophages and lytic bacteria.

*R. megarrhizum* is an obligate parasite that belongs to the phylum Chytridiomycota. It is specific to *Planktothrix* [10] and utilizes chemotactic zoospores to trace its host [25]. A single zoospore can infect several cells, which are killed within days. Infection can burst into epidemics that can devastate entire *Planktothrix* populations [10],[35]. The parasite was directly detected in Lake Steinsfjorden in 1997–1999 [10]. Its long-term presence was verified by sediment DNA analysis for the years 1959–1973 and 1995–2013 [33]. The ciliate *N. ornata* is specialized in *Planktothrix* and other filamentous cyanobacteria [36]. Its unique morphology allows the microbe to ingest and digest *Planktothrix* filaments that are much longer than the ciliate itself [37]. Laboratory and field studies showed that *N. ornata* has the potential to keep *Planktothrix* under tight control [34],[36].

Laboratory experiments of the present study included two strains of each, *Planktothrix*, *R. megarrhizum* and *N. ornata*. *Planktothrix* NIVA-CYA98 and NIVA-CYA532 were isolated from Steinsfjorden in 1982 and 2008, respectively. They differ in content of bioactive compounds [38], gene sequences [38]–[41] and interaction with *R. megarrhizum* [10]. *R. megarrhizum* Kol2008 and Lys2009 were isolated from lakes in close proximity to Steinsfjorden in 2008 and 2009, respectively. Their presence in Lake Steinsfjorden was verified by strain-specific PCR for the years 1997–1999 [10]. *N. ornata* Cil3 and Cil4 were isolated from Lake Steinsfjorden in 2016.

The *Planktothrix* population of Lake Steinsfjorden consists of a phycoerythrin-producing, red colored variant and a phycoerythrin-lacking, green variant. According to their morphology, these variants would typically be assigned to *Planktothrix rubescens* and *P. agardhii*, respectively. However, red and green *Planktothrix* from Lake Steinsfjorden overlap in chemotaxonomic and genetic markers [38] and in interaction with *R. megarrhizum* [10]. Both *Planktothrix* variants form metalimnetic blooms and grow best under low light and low temperature conditions, i.e., both show growth pattern that are typical for *P. rubescens* [28]. Moreover, a recent study showed that horizontal transfer of phycoerythrin genes had happened in Lake Steinsfjorden, converting green *Planktothrix* into the functional red variant [41]. Overall, it is likely that red and green *Planktothrix* in Lake Steinsfjorden are closely related and that the local green variant is rather a “green *P. rubescens*” than a typical *P. agardhii*. For the present study, both red and green *Planktothrix* were therefore treated as conspecific and referred to as *Planktothrix*.

### 2.2. Growth of Planktothrix as a function of temperature

NIVA-CYA98 and NIVA-CYA532 were cultured in 250 ml flasks with continuous aeration and illumination by diluting them to an optical density of 0.06 (5 cm cuvette, 800 nm) every 2^nd^ day. Sterile BG11 served as culture medium. To study *Planktothrix* growth as a function of temperature, cultures were run at 10, 12, 14, 16, 18 and 20 °C at 12 µmol photons m^−2^ s^−1^. These conditions reflect a typical situation that NIVA-CYA98 and NIVA-CYA532 experience in their natural environment during summer stratification [28]. Once cultures had reached steady state, their specific growth rates were determined for a period of 10 days, using measurements of optical density as basis.

### 2.3. Growth of N. ornata as function of temperature and food source

Cil3 and Cil4 were cultured in 50 ml glass vessels using *Planktothrix* NIVA-CYA97/1 (maintained in BG11 medium at 18 °C and at 5 µmol photons m^−2^ s^−1^) as food. Growth experiments with Cil3 and Cil4 utilized material from the above steady state NIVA-CYA98 and NIVA-CYA532 cultures. The experiments were conducted in 10 ml glass tubes, containing 2 ml *Planktothrix* suspension in sterile BG11 with an optical density of 0.03 and 4 ciliates each. The tubes were incubated at the same temperatures and irradiance as the *Planktothrix* cultures used in the tests were adapted to. Ciliates were counted at low magnification after 48 hours and the results were used to calculate the specific growth rate. Tests were run in quadruplicates.

### 2.4. Prevalence of R. megarrhizum infection as a function of temperature and host identity

Chy-Kol2008 and Chy-Lys2009 were cultured as described earlier [7]. Both strains propagate with zoospores that are formed in epiphytic sporangia [42]. Zoospore suspensions were produced by filtering *R. megarrhizum* cultures over 10 µm gauze. Zoospore density was determined with a hemocytometer after fixation with Lugol's solution. Infection experiments utilized material from the above steady state NIVA-CYA98 and NIVA-CYA532 cultures. The experiments were conducted in 10 ml glass tubes filled with 2 ml test suspension in sterile BG11. The final *Planktothrix* density corresponded to an optical density of 0.03, while that of *R. megarrhizum* zoospores was 40000 ml^−1^. The glass tubes were incubated at the same temperatures and irradiance as the *Planktothrix* cultures used in the experiments were adapted to. After 48 hours, the prevalence of *R. megarrhizum* infection, defined as % host filaments infected, was determined by light microscopic inspection of 100 *Planktothrix* filaments per replicate. Only filaments carrying at least one epiphytic sporangium were counted as successfully infected. Tests were run in quadruplicates.

### 2.5. Identification of thermal refuges using the output of laboratory experiments

The specific growth rates of *Planktothrix* and *N. ornata* strains and the prevalence of *R. megarrhizum* infection were plotted against temperature. The resulting relationships were fitted to appropriate mathematical models. Zero points of the models were used to identify ranges of temperature that allow *Planktothrix* to grow while suppressing *R. megarrhizum* or *N. ornata* strains. This information was used to identify environmental refuges that protect *Planktothrix* from all *R. megarrhizum* or *N. ornata* strains.

### 2.6. Thermal refuges and Planktothrix bloom formation in Lake Steinsfjorden

The occurrence of thermal refuges in Lake Steinsfjorden, the access of *Planktothrix* to these refuges and the consequences for bloom formation were studied retrospectively using monitoring data from the period 1998–2004. These data were collected by the Norwegian Institute for Water Research and are freely available [28],[43].

The monitoring data and the output of above described laboratory experiments were used to identify water layers in Lake Steinsfjorden with refuge temperatures. The position of such layers was then compared graphically with the position of *Planktothrix* in the water column to test whether the cyanobacterium had access to thermal refuges in nature.

Bloom formation occurs when the net growth rate of *Planktothrix* is positive for a longer period of time. Hence, to promote bloom formation thermal refuges must support positive *Planktothrix* net growth. To test for this, net growth rates of *Planktothrix* for consecutive sampling dates were calculated using cumulative abundances in the entire water column as input. Net growth rates were then plotted against the mean temperature that *Planktothrix* experienced. This parameter was determined as the weighted mean of temperatures measured throughout the water column on two consecutive sampling dates. The also available measurements of the *Planktothrix* biomass concentration at all depths served as weights. The resulting relationship between net growth rate and temperature was fitted to a suitable polynomic model, which was then used to test whether refuge temperatures indeed support positive net growth of the Steinsfjorden *Planktothrix* population.

*Planktothrix* bloom formation in Lake Steinsfjorden is best documented for summer stratification [28]. It was therefore tested how temperature impacts the probability of summer bloom formation by *Planktothrix*. The mean temperature that *Planktothrix* experienced during a given summer was determined as the weighted mean of temperatures measured throughout the water column in June–August. The also available measurements of the *Planktothrix* biomass concentration at all depths and sampling dates served as weights. The temperature that *Planktothrix* experienced during a given summer was plotted against the cumulative abundance in the entire water column that *Planktothrix* reached at the end of August. The resulting relationship was subjected to linear regression analysis.

## 3. Results

### 3.1. The impact of temperature on Planktothrix and on its microbial antagonists

**Figure 1.**
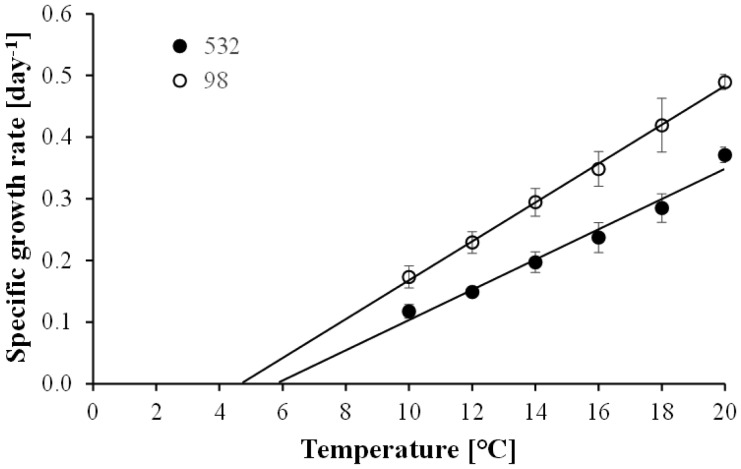
The effect of temperature on the specific growth rate of *Planktothrix* NIVA-CYA98 and NIVA-CYA532. Data points represent mean values of four replicates ± standard deviation. The relationships were fitted to linear models (NIVA-CYA98: 0.03*temperature + 0.15, R > 0.99, P < 0.001; NIVA-CYA532: 0.02*temperature + 0.14, R = 0.98, P < 0.001).

**Figure 2.**
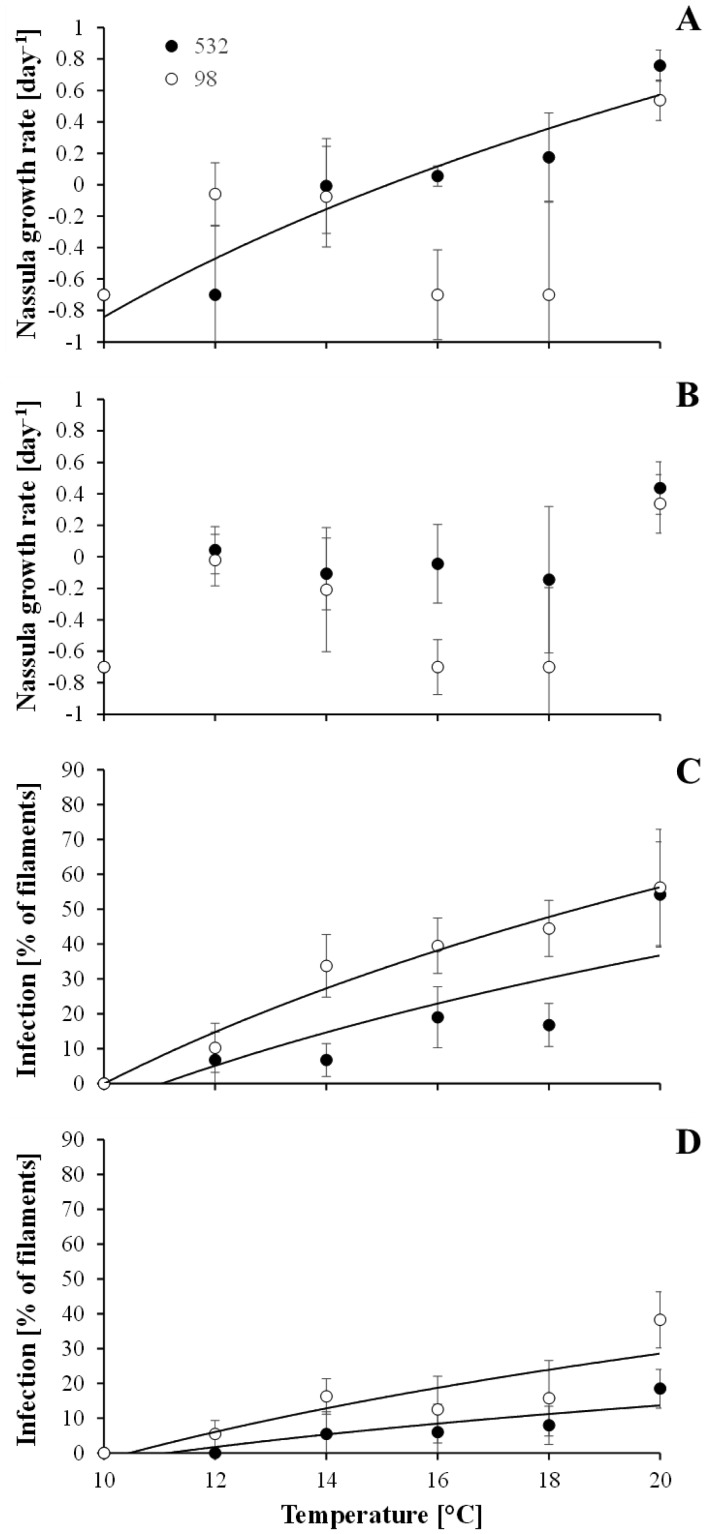
A and B: Effect of *Planktothrix* NIVA-CYA98 and NIVA-CYA532 and temperature on the specific growth rate of *N. ornata* Cil3 (A) or Cil4 (B). Data points represent mean values of four replicates ± standard deviation. The specific growth rate of Cil3 increased with temperature when fed NIVA-CYA532 (2.04*ln[temperature] − 5.54, R = 0.94, P < 0.01). Regression analysis did not return mathematical models for other *Planktothrix-N. ornata* combinations. C and D: Effect of *Planktothrix* NIVA-CYA98 and NIVA-CYA532 and temperature on the prevalence of *R. megarrhizum* infection after 2 days of exposure to Kol2008 (C) or Lys2009 (D). Data points represent mean values of four replicates ± standard deviation. The relationships could be fitted to logarithmic models (NIVA-CYA98 vs. Kol2008: 81.4*ln[temperature] − 187.4, R = 0.98, P < 0.001; NIVA-CYA532 vs. Kol2008: 61.9*ln[temperature] − 148.8, R = 0.82, P < 0.05; NIVA-CYA98 vs. Lys2009: 44.0*ln[temperature] − 103.3, R = 0.87, P < 0.05; NIVA-CYA532 vs. Lys2009: 23.4*ln[temperature] − 56.3, R = 0.89, P < 0.05).

The specific growth rate of *Planktothrix* NIVA-CYA98 and NIVA-CYA532 increased linearly with temperature (Figure 1). The lower limit for growth was estimated to 4.7 or 5.8 °C, depending on the *Planktothrix* strain that was tested. In most cases, *N. ornata* did not grow at temperatures below 20 °C (Figure 2A, B). The only exception was when the strain Cil3 was fed with *Planktothrix* NIVA-CYA532. Here the growth rate of the ciliate increased with temperature and regression analysis suggested that positive growth started at 15.8 °C. However, at 20 °C the growth rate of *N. ornata* was similar to or even higher than that of *Planktothrix*. The prevalence of *R. megarrhizum* infection after 2 days increased with temperature to reach a maximum of more than 50% at 20 °C (Figure 2C, D). The relationship between temperature and prevalence of infection after 2 days could be described with a logarithmic function, projecting the lower temperature limit for infection to be 10.0–11.1 °C.

### 3.2. A thermal refuge for Planktothrix

*Planktothrix* had a broader temperature tolerance range than its microbial antagonists (Figures 1 and 2). Considering only the most thermophilic *Planktothrix* strain and the most cold tolerant *R. megarrhizum* strain, *Planktothrix* could grow without risking parasite infection between 5.8 and 10 °C. *N. ornata* was even more thermophilic than *R. megarrhizum*, allowing *Planktothrix* to grow undisturbed by the ciliate between 5.8 and 15.8 °C. These data therefore suggest that there is a thermal refuge at 5.8–10 °C that allows all *Planktothrix* strains to grow without being exploited by *R. megarrhizum* or *N. ornata*.

### 3.3. The thermal refuge and Planktothrix bloom formation in Lake Steinsfjorden

**Figure 3.**
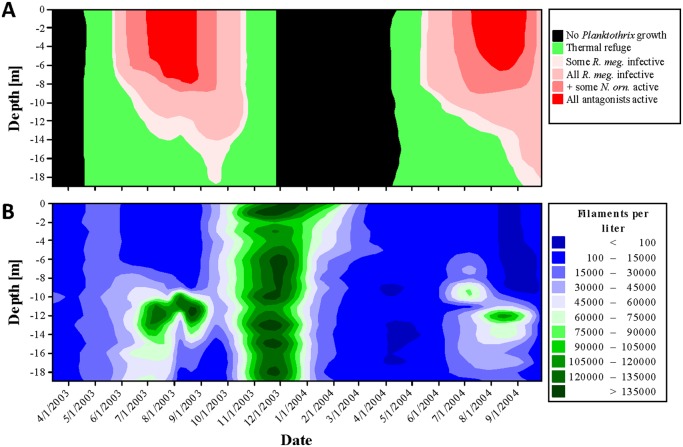
A: Laboratory experiments of the present study provided information on temperature tolerance ranges of *Planktothrix* and its microbial antagonists. These data and temperature profiles taken in Lake Steinsfjorden in 2003/2004 were used to identify water layers with temperatures that (black) prevent *Planktothrix* growth, (green) allow *Planktothrix* growth without microbial exploitation and (light to dark red) expose *Planktothrix* to an increasing exploitation by *R. megarrhizum* and later by *N. ornata*. Temperature profiles were collected by the Norwegian Institute for Water Research and are freely available [28],[43]. B: Depth distribution of *Planktothrix* in 2003/2004. Data were collected by the Norwegian Institute for Water Research and are freely available [28],[43].

**Figure 4.**
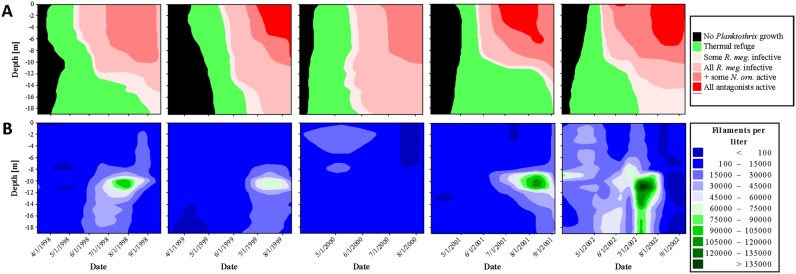
The same as in Figure 3 but for the years 1998–2002.

Refuge temperatures probably allowed *Planktothrix* to grow without being exploited by *R. megarrhizum* and *N. ornata* during spring and autumn circulation (Figures 3 and 4). This was also true for summer stratification, but only in the lower part of the metalimnion and only until warmer water was mixed into deeper parts of the water column. During winter, very low temperatures probably protected *Planktothrix* from *R. megarrhizum* and *N. ornata*, while they also denied *Planktothrix* to grow (Figure 3).

Its buoyancy regulation system helped *Planktothrix* to exploit refuge temperatures. This was most obvious during summer stratification when *Planktothrix* moved into those parts of the metalimnion where temperature conditions probably allowed the cyanobacterium to grow with a minimum of exploitation by *R. megarrhizum* and *N. ornata* (Figures 3 and 4). This was typically associated with an increase in *Planktothrix* abundance, often followed by a sharp decline in August or September when *Planktothrix* came in contact to warmer water. *Planktothrix* was less successful in years with a warmer than usual metalimnion (1999, 2000, 2002; Figure 4). Monitoring data covering autumn full circulation were only available for 2003. In this year, low temperatures throughout the water column were associated with a massive *Planktothrix* bloom (Figure 3).

**Figure 5.**
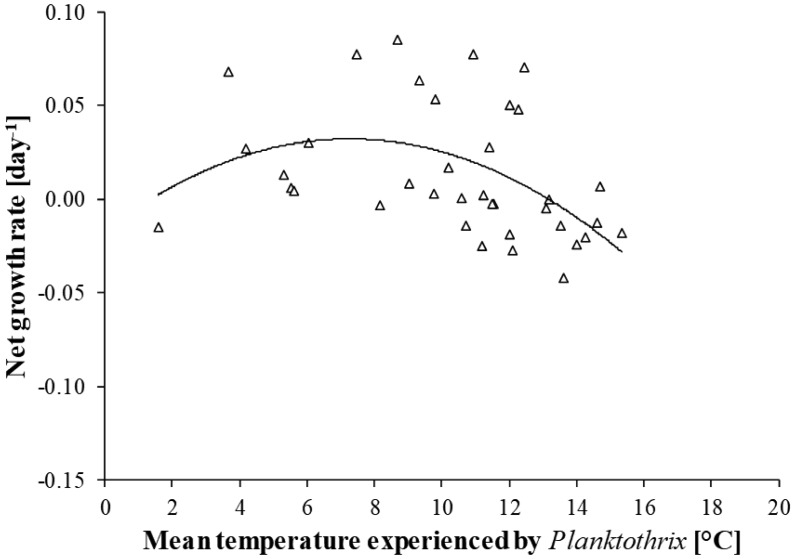
Effect of temperature that *Planktothrix* experienced in Lake Steinsfjorden in 1998–2004 on the *Planktothrix* net growth rate. The relationship could be fitted to a 2^nd^ order polynomic function (−0.0009*[temperature]^2^ + 0.0134*temperature − 0.0165, R = 0.47, P < 0.01).

**Figure 6.**
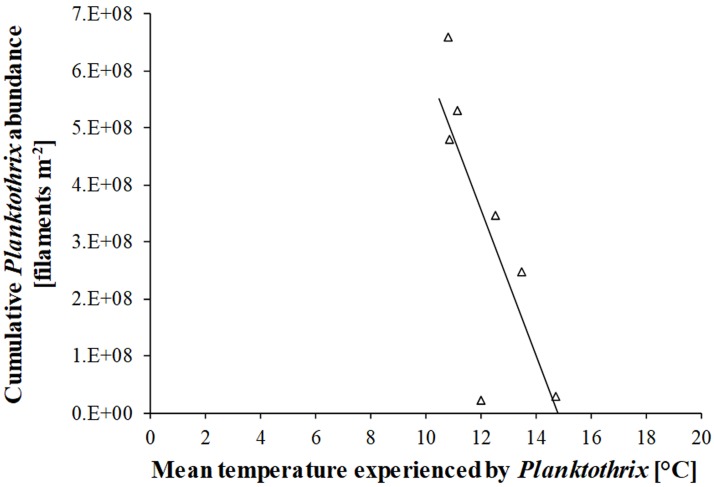
Effect of temperature that *Planktothrix* experienced in Lake Steinsfjorden during summers in 1998–2004 on the cumulative *Planktothrix* abundance in the entire water column at the end of each summer. The relationship could be fitted to a linear model (−1e08*temperature + 2e09, R = 0.77, P < 0.05).

In 1998–2004, the net growth rate of *Planktothrix* was almost always positive between 2 and 10 °C and there was an increasing trend to negative values when *Planktothrix* experienced higher temperatures (Figure 5). The relationship between the net growth rate of *Planktothrix* and the mean temperature that the cyanobacterium experienced could be fitted to a 2^nd^ order polynomic function, suggesting that the net growth rate of *Planktothrix* is positive between 2 and 13 °C. The cumulative abundance that *Planktothrix* reached at the end of a summer and the mean temperature that *Planktothrix* experienced during that summer were negatively correlated (Figure 6). The resulting regression line suggests that the probability of *Planktothrix* summer bloom formation is highest below 11 °C and lowest at temperature higher than 15 °C.

## 4. Conclusions

In laboratory experiments, the temperature range 5.8–10 °C formed a thermal refuge that enabled *Planktothrix* to grow without being exploited by *R. megarrhizum* and *N. ornata*. In Lake Steinsfjorden, access to this refuge was associated with positive net growth and with a high probability of summer *Planktothrix* bloom formation, indicating that refuge temperatures indeed allow *Planktothrix* to grow without suffering a significant loss of biomass due to antagonistic biological interactions. Laboratory experiments also revealed that above 10 °C exploitation of *Planktothrix* by multiple microbes increases in a temperature-dependent manner to reach a considerable strength. This increase in microbial exploitation is likely to rapidly offset the stimulating effect of warm water on *Planktothrix* growth, which explains why contact to warm water of the *Planktothrix* population in Lake Steinsfjorden was associated with decreases in net growth rate and in probability of summer bloom formation as well as with population collapses. That these negative effects on the local *Planktothrix* population were indeed caused by a temperature-driven increase in microbial exploitation is supported by the observation of an earlier study in Lake Steinsfjorden, which detected *R. megarrhizum* infection mainly towards the end of summer stratification, when *Planktothrix* was mixed into layers with warm surface water [10]. Similar observations were made by Davis and coworkers in Lake Blelham Tarn (UK) [35]. Above findings and observations therefore suggest that differences in temperature requirements between *Planktothrix* and its microbial antagonists bring about a thermal refuge at low temperatures that allows the cyanobacterium to form blooms despite growing under rather suboptimal thermal conditions.

The proposed thermal refuge at low temperatures is a result of *R. megarrhizum* and *N. ornata* being more thermophilic than their cyanobacterial prey. This may not be an unusual situation. Many studies showed that cyanophages, lytic bacteria and heterotrophic flagellates also can be more thermophilic than their cyanobacterial prey [23],[44]–[48]. It seems therefore possible that the occurrence of thermal refuges might be a hitherto largely overlooked but nevertheless widespread cause of bloom formation in cyanobacteria in general. Moreover, studies on parasitism in the diatom *A. formosa* found indications of refuges at low irradiances and comparatively high temperatures [18]–[21],[49]. It is unknown whether such refuges also occur in biological interactions involving cyanobacteria. But if they do, these refuges may well explain why cyanobacteria form blooms over a wide range of environmental conditions [1],[2],[50]. Here it must also be considered that cyanobacteria can reduce microbial exploitation by intraspecific diversification [51]. Bloom formation may therefore result from access to environmental refuges and/or from intraspecific diversification. Besides, cyanobacterial defense systems may also play a role [6]–[8].

In Lake Steinsfjorden, *Planktothrix* actively moved into those parts of the metalimnion that had refuge or near-refuge temperatures. This might be interpreted as behavioral defense against microbial exploitation. Usually, the metalimnetic stratification of *Planktothrix* is seen as a way to gain access to nutrients from deeper parts of the water column without leaving the euphotic zone [28]. This is certainly correct. But here it is important to recognize that by moving into the metalimnion *Planktothrix* exposes itself to temperatures that only support low growth rates. It seems unlikely that such slow growth would be enough to compensate for the mortality inflicted by microbial antagonists. A metalimnetic stratification would therefore be of no real advantage as long as it gives access to nutrients without also providing protection from microbial exploitation. I thus suggest that the metalimnetic stratification of *Planktothrix* has evolved as a way to simultaneously gain access to nutrients and to a thermal refuge.

According to common belief, cyanobacterial bloom formation is a result of favorable growth conditions [1]. Above findings suggest that this is not always the case. Suboptimal growth conditions such as low temperatures can moderate or even halt microbial exploitation as they did in the case of *Planktothrix* in Lakes Steinsfjorden. This limits the loss of biomass, which increases the probability of bloom formation even at low growth rates. Optimal growth conditions, on the other hand, can expose cyanobacteria to multiple microbial antagonists that may inflict considerable mortality on their cyanobacterial prey. Bloom formation under these conditions may require defensive traits or intraspecific diversification to limit the loss of biomass caused by microbial exploitation.
